# Methane, Bacteria, Fungi, and Fermentation: Pathophysiology, Diagnosis and Treatment Strategies for Small Intestinal Bacterial Overgrowth, Intestinal Methanogen Overgrowth and Small Intestinal Fungal Overgrowth

**DOI:** 10.3390/cimb47090713

**Published:** 2025-09-02

**Authors:** Adam Wawrzeńczyk, Marta Czarnowska, Samira Darwish, Aleksandra Ćwirko-Godycka, Kinga Lis, Maciej Szota, Paweł Treichel, Aleksandra Wojtkiewicz, Katarzyna Napiórkowska-Baran

**Affiliations:** 1 Department of Allergology, Clinical Immunology and Internal Diseases, Collegium Medicum Bydgoszcz, Nicolaus Copernicus University Toruń, 85-067 Bydgoszcz, Poland; adam.wawrzenczyk@cm.umk.pl (A.W.); kinga.lis@cm.umk.pl (K.L.); maciejszota98@gmail.com (M.S.); 2 Student Research Club of Clinical Immunology, Department of Allergology, Clinical Immunology and Internal Diseases, Collegium Medicum Bydgoszcz, Nicolaus Copernicus University Toruń, 85-067 Bydgoszcz, Poland; marta.czarnowska.med@gmail.com (M.C.); samiradarwish00@gmail.com (S.D.); aleksandragood7@gmail.com (A.Ć.-G.); treichel.pawel@gmail.com (P.T.); wojtkiewiczaleksandra16@gmail.com (A.W.)

**Keywords:** gastrointestinal dysbiosis, small intestinal bacterial overgrowth (SIBO), intestinal methanogen overgrowth (IMO), small intestinal fungal overgrowth (SIFO), large intestinal bacterial overgrowth (LIBO)

## Abstract

The human gastrointestinal tract hosts a complex ecosystem known as the gut microbiota, which plays a crucial part in digestion and immune system function. Among the clinically recognized manifestations of dysbiosis in this system are Small Intestinal Bacterial Overgrowth (SIBO), Intestinal Methanogen Overgrowth (IMO), Small Intestinal Fungal Overgrowth (SIFO), and Large Intestinal Bacterial Overgrowth (LIBO). This study aims to investigate the complex pathophysiological mechanisms underlying these syndromes and their diagnostics and therapeutic options, focusing primarily on the roles of methane-producing archaea and fungal overgrowth. The methods employed in this study involve a comprehensive analysis and synthesis of peer-reviewed articles, systematic reviews, clinical trials, and meta-analyses. This review summarizes that methane production by *Methanobrevibacter smithii* was linked to altered fermentation, reduced microbial diversity, and slowed intestinal transit. Fungal species were associated with increased intestinal permeability, inflammation, and biofilm formation. Targeted interventions addressing microbial imbalances demonstrated potential therapeutic value. This review highlights the complex and multifactorial nature of gut dysbiosis, revealing its impact beyond the gastrointestinal tract. While emerging therapies targeting methanogens, fungi, and biofilms show promise, further research is essential to optimize their clinical application. The findings emphasize the need for interdisciplinary collaboration to refine diagnostic and therapeutic strategies.

## 1. Gastrointestinal Dysbiosis Overview

The gut microbiota, a complex ecosystem of microorganisms, plays a vital role within the human gastrointestinal tract in digestion, immune modulation, and the maintenance of intestinal homeostasis. In recent years, growing attention has been directed toward gastrointestinal dysbiosis as a disruption of this microbial balance, which has been linked to a variety of gastrointestinal and systemic conditions. Clinically recognized forms of dysbiosis include Small Intestinal Bacterial Overgrowth (SIBO), Intestinal Methanogen Overgrowth (IMO), Small Intestinal Fungal Overgrowth (SIFO), and Large Intestinal Bacterial Overgrowth (LIBO), although the last one remains less characterized in the literature and lacks standardized diagnostic criteria [1,2,3,4].

Understanding gastrointestinal dysbiosis requires analyzing the complex imbalances within the microbiota and their clinical consequences. These conditions are multifactorial and present significant clinical challenges. Methanogenic archaea, such as *Methanobrevibacter smithii*, have been associated with altered fermentation, decreased microbial diversity, and delayed intestinal transit [3,5]. Fungal overgrowth, in turn, has been linked to increased intestinal permeability, chronic inflammation, and biofilm formation [3]. These findings highlight the need for precise diagnostic tools and targeted therapies that address specific microbial imbalances.

This review aims to synthesize current knowledge about the mechanisms driving gut dysbiosis, with particular focus on the roles of methanogens and fungi, and to present available diagnostic methods and therapeutic options. Only through a comprehensive understanding of physiological and pathophysiological relationships is it possible to achieve a clearer picture of the clinical manifestations and guide appropriate therapeutic interventions that emerge later in the text [1,2].

## 2. Small Intestinal Bacterial and Methanogen Overgrowth

The clinical criteria for diagnosing Small Intestinal Bacterial Overgrowth (SIBO) and Intestinal Methanogen Overgrowth (IMO) remain a subject of debate, complicating standardization efforts within clinical practice [6]. SIBO is typically identified by the presence of ≥10^3^ colony-forming units (CFU) per milliliter in duodenal or jejunal aspirates. However, some research suggests that a threshold of ≥10^5^ CFU/mL may provide a more accurate diagnostic cutoff, reflecting the variability in microbial concentrations among individuals [7,8]. This discrepancy reveals the challenges healthcare professionals face in setting universally accepted parameters for diagnosis [6,7].

Methanogen overgrowth, commonly driven by *Methanobrevibacter smithii*, is associated with methane production, which has a well-documented inhibitory effect on gut motility [4,6,8]. This alteration in motility is a significant distinguishing feature of IMO, as it often manifests with constipation-predominant symptoms, further complicating its differentiation from other dysbiosis-related disorders [4,8]. The ongoing refinement of diagnostic criteria underscores the necessity for continued research and consensus-building within the scientific community [6,7].

The prevalence rates of SIBO and IMO vary widely among populations, highlighting the multifactorial and heterogeneous nature of these conditions [3,9]. In individuals with irritable bowel syndrome (IBS), particularly those experiencing constipation-predominant IBS (IBS-C), the overlap with SIBO and IMO is well established [4,9]. For instance, approximately 36.7% of IBS patients are diagnosed with SIBO, and of these, 25.3% demonstrate methane positivity, enabling the differentiation of IMO from hydrogen-dominated SIBO [9]. Further studies indicate that methane production contributes significantly to symptomatology in these cases, as nearly 30% of SIBO cases involve methanogen overgrowth. These findings emphasize the necessity of methane detection in accurately diagnosing IMO, particularly in patients whose chief complaint is constipation [3,9]. Such data reinforce the importance of tailored diagnostic and therapeutic approaches to address the unique pathophysiology presented by individual patients [3,4,9].

The development of SIBO and IMO is influenced by a range of risk factors, including impaired gastric motility, anatomical abnormalities, and the use of proton pump inhibitors (PPIs). PPIs are particularly significant because they reduce gastric acidity, disrupting gut homeostasis by impairing the natural defense mechanisms against bacterial migration into the small intestine [9,10]. Systemic diseases, such as diabetes and hypothyroidism, further predispose individuals to dysbiosis by altering intestinal motility and compromising immune functions [3,9]. Patients with intestinal failure who rely on parenteral nutrition experience disproportionately high rates of SIBO, underscoring the interplay between underlying conditions and microbial imbalances [11]. This situation occurs due to several physiological disruptions. First, the absence of enteral stimulation leads to mucosal atrophy, creating favorable conditions for bacterial overgrowth. Second, TPN alters the composition of gut microbiota by depriving the intestinal lumen of nutrients, which can disrupt microbial balance. Additionally, TPN is associated with increased intestinal permeability, compromising the gut barrier and promoting bacterial translocation. Finally, prolonged TPN use can impair gut-associated immune function, reducing local defense mechanisms and further predisposing the intestine to microbial overgrowth [10,12,13]. These combined effects underscore the importance of careful monitoring and, when feasible, the inclusion of minimal enteral feeding to maintain gut integrity [10].

Additionally, lifestyle factors such as unhealthy dietary patterns, obesity, and metabolic syndrome exacerbate this disruption, illustrating the complex interaction between systemic metabolic disorders and gastrointestinal health. Addressing these risk factors holistically is essential for a comprehensive understanding of dysbiosis and its treatment [3,10,11].

*Helicobacter pylori* (*H. pylori*), a common gastric pathogen, has been increasingly explored in relation to SIBO, given its impact on gastric acid secretion and gastrointestinal motility. Alterations in gastric acidity and mucosal immunity caused by *H. pylori* infection may create a favorable environment for bacterial overgrowth in the small intestine, suggesting a potential link between these two conditions. Furthermore, meta-analysis conducted by Xiong et al. has shown that *H. pylori* can influence gut function through the brain–gut axis and by modulating gastrointestinal hormones, such as gastrin and cholecystokinin, leading to symptoms like abdominal pain and diarrhea.

Moreover, in a meta-analysis, Liao at al. have shown that, compared to adults without HP infection, those with HP infection were associated with a higher prevalence of SIBO. The association between HP infection and SIBO was stronger in younger than older patients [14]. SIBO is often associated with factors such as prolonged use of PPIs, which reduce gastric acid secretion and promote bacterial colonization in the upper GI tract. *H. pylori* infection may coexist with SIBO, and its treatment can influence gut microbial balance. Therapeutic implications require distinguishing between the treatment of *H. pylori* and SIBO. The study by Konrad et al. demonstrated that the combination of amoxicillin and rifaximin achieved a 64% eradication rate of *H. pylori*, suggesting potential benefit with extended therapy [15]. This combination appears particularly effective in patients with concurrent SIBO, leading to symptom improvement. Due to rising resistance to metronidazole, alternatives like clarithromycin, tetracycline, and levofloxacin are increasingly used, though with limited success. Rifaximin remains the most effective and well-tolerated option for SIBO management according to current evidence [16]. Eradication therapy not only improves symptoms in *H. pylori*-positive IBS patients but also alters the gut microbiota—typically decreasing *Escherichia coli* abundance while increasing *Klebsiella* and other Enterobacteriaceae—suggesting a broader impact on intestinal microbial balance [17].

The clinical presentations of SIBO and IMO frequently overlap, complicating differential diagnosis and treatment strategies. SIBO often manifests as bloating, diarrhea, and abdominal pain, while IMO is predominantly associated with constipation [18,19]. For example, bloating occurs in 70.4% of patients with hydrogen-positive SIBO, whereas constipation is reported in 43.9% of methane-positive IMO cases, reflecting methane’s distinct role in altering intestinal function [18]. These overlapping symptoms pose challenges for clinicians, emphasizing the need for reliable diagnostic tools to differentiate between these related conditions. This complexity underscores the importance of symptom-specific investigations to ensure precise and effective therapeutic intervention [8,18].

Hydrogen and methane breath tests are widely used for diagnosing SIBO and IMO due to their non-invasive nature and accessibility. A rise in hydrogen levels of ≥20 parts per million (ppm) above baseline following carbohydrate ingestion indicates SIBO, while methane levels of ≥10 ppm suggest IMO [4,6,20]. Despite their widespread applicability, these tests face considerable limitations regarding sensitivity and specificity. For instance, glucose breath tests demonstrate sensitivity ranging from 52% to 63%, whereas lactulose breath tests have an even broader range of 31% to 68%. These variations raise concerns about their reliability, particularly for detecting overgrowth in the distal small intestine. The technical limitations of these diagnostic measures point to the need for complementary approaches to improve diagnostic accuracy and clinical decision-making [4,6,7,20].

Given the limitations of breath testing, alternative diagnostic methods warrant exploration to enhance clinical precision. Small bowel aspirates and cultures provide a more definitive diagnostic approach by directly quantifying bacterial populations in the small intestine, although their invasive nature limits routine use [21]. Emerging molecular diagnostic techniques, such as next-generation sequencing and stool metagenomics, offer considerable promise in addressing these gaps by enabling comprehensive microbial profiling [8,10,22]. These tools can identify specific bacterial and methanogen species implicated in dysbiosis conditions like SIBO and IMO [8,10]. Collaborative efforts among gastroenterologists and researchers are critical to developing integrated diagnostic frameworks that combine traditional and innovative modalities for improved patient outcomes [8,10,22].

In summary, the diagnosis and management of SIBO and IMO involve navigating complex and often overlapping clinical presentations, influenced by diverse risk factors and diagnostic limitations. The interplay between evolving diagnostic criteria, symptom heterogeneity, and emerging molecular tools underscores the importance of a multi-faceted approach to these conditions.

## 3. Key Findings

Methanogenic archaea, such as *Methanobrevibacter smithii*, are particularly significant due to their role as hydrogen sinks. By consuming hydrogen to produce methane, these archaea alter the fermentation dynamics within the gut microbiome. This reduction in hydrogen availability impacts the growth of hydrogen-dependent bacteria, potentially skewing the microbial population balance [23,24]. This metabolic advantage allows methanogens to outcompete other microorganisms, exacerbating microbial imbalances and leading to conditions like IMO [23]. Furthermore, this altered fermentation dynamic contributes to the accumulation of short-chain fatty acids (SCFAs), which, in combination with methane production, can cause symptoms such as bloating and abdominal discomfort [3]. Such insights highlight the critical role of methanogens in disrupting gut homeostasis [24].

The slowing of the intestinal transit in constipation-dominant conditions is one of the hallmark features distinguishing methane-dominant dysbiosis from other types, such as hydrogen-producing bacterial overgrowths, which are often associated with diarrhea [22,24]. Moreover, methane levels have been shown to correlate with symptom severity in conditions like IMO, emphasizing its clinical relevance [22,23]. This underscores the importance of methane-targeting therapeutic strategies to alleviate symptoms and restore normal gut functioning [23].

The unique metabolic pathways that allow methanogenic archaea to thrive in anaerobic environments give them a notable advantage in conditions of dysbiosis [24,25]. By efficiently consuming hydrogen and producing methane, they create an environment less conducive to the survival of competing microorganisms, thereby reducing microbial diversity and exacerbating dysbiosis. This dominance of methanogens further perpetuates the imbalances in microbial populations, making their targeting a key therapeutic focus [24]. This competitive edge over other gut microbes accentuates the cyclical nature of dysbiosis and underscores the necessity of addressing methanogens’ metabolic processes to re-establish microbial equilibrium and alleviate associated symptoms [25].

Methanogen activity also indirectly affects the production of SCFAs, critical byproducts of microbial fermentation in the gut. The altered fermentation process resulting from reduced hydrogen availability due to methanogenesis leads to an accumulation of SCFAs, which can influence various aspects of gut physiology. While SCFAs are generally considered beneficial in maintaining gut health, the excessive presence of SCFA-producing bacteria in the context of dysbiosis can exacerbate bloating and other gastrointestinal symptoms, further complicating the clinical presentation of conditions like IMO [3,26]. This intricate interplay between methanogenesis and SCFA production highlights the broader systemic effects of microbial imbalances and the need for precision therapies targeting these mechanisms [24].

Therapeutic interventions aimed at methanogens have demonstrated potential in alleviating dysbiosis-related symptoms by disrupting methane production and restoring microbial balance. For instance, reducing populations of *Methanobrevibacter smithii* has been associated with improved gut motility and alleviation of constipation in patients with conditions like IBS-C and IMO [23]. The potential to manipulate microbial populations and their metabolic activities through targeted treatments offers hope for more effective management of dysbiosis syndromes [23,24]. These strategies underscore the importance of personalized medicine approaches that consider the unique microbial compositions of individual patients [23].

Fungal overgrowth, particularly involving species such as *Candida albicans*, introduces additional layers of complexity to dysbiosis syndromes. Fungi contribute to gut inflammation by compromising the intestinal epithelial barrier, thereby facilitating the translocation of pathogens, antigens, and toxins into the systemic circulation. However, this phenomenon is not unique to fungal proliferation; similar barrier dysfunction may arise from bacterial overgrowth or the effects of microbial metabolites. Markers of gut barrier integrity reflect both enterocyte injury and tight junction dysfunction. Intestinal fatty acid-binding protein (I-FABP) indicates enterocyte damage, whereas claudin-3 (detected in urine) and zonulin (detected in blood) are markers of tight junction disruption associated with increased intestinal permeability (“leaky gut”) [25].

In the small intestine, dysmotility, bacterial overgrowth, dysbiosis, and epithelial barrier dysfunction are interrelated and collectively contribute to the development of “leaky gut”, which permits the translocation of various microbial products. These include lipopolysaccharide (LPS), a key endotoxin of Gram-negative bacteria, as well as peptidoglycans, lipopeptides, and bacterial DNA from both Gram-positive and Gram-negative bacteria, along with fungal components. Such translocated products interact with Toll-like receptors (TLRs) on immune and other host cells, triggering innate immune responses and promoting proinflammatory signaling cascades.

This leads to elevated levels of cytokines such as IL-1β and TNF-α, exacerbates gastrointestinal symptoms, and contributes to systemic complications [27]. The inflammatory response associated with fungal dysbiosis represents a significant pathological mechanism in syndromes like small intestinal fungal overgrowth (SIFO), highlighting the need for precise diagnostic and therapeutic strategies [25].

Increased intestinal permeability, or “leaky gut,” is a hallmark feature of excessive fungal proliferation and is often driven by the ability of fungi, such as *Candida albicans*, to form hyphae and biofilms. These structures physically damage the intestinal mucosa, increasing permeability and allowing harmful substances to cross the epithelial barrier. The resulting systemic inflammation exacerbates gastrointestinal and extraintestinal symptoms, demonstrating the far-reaching effects of excessive fungal growth. Addressing these mechanisms through antifungal therapies and biofilm disruptors is crucial for restoring mucosal integrity and improving outcomes in patients with fungal dysbiosis [28].

The interaction between fungal and bacterial populations further amplifies the pathogenicity of dysbiosis syndromes. For example, biofilm formation resulting from interactions between *Candida albicans* and bacteria such as *Escherichia coli* creates a resilient microenvironment that enhances resistance to antimicrobial treatments [6]. These biofilms not only protect microbial communities but also exacerbate the severity of gastrointestinal conditions, complicating treatment strategies. This underscores the need for therapeutic approaches that target multi-species biofilms to effectively address fungal and bacterial overgrowth [29].

Alterations in fungal communities also have systemic implications, as evidenced by studies associating elevated fungal colonization with increased systemic markers of inflammation.

These findings link fungal dysbiosis with inflammatory conditions beyond the gastrointestinal tract, including neuropsychiatric disorders. Such systemic effects emphasize the broader significance of fungal contributions to health and the need for comprehensive strategies to address their pathogenicity. This further solidifies the rationale for antifungal therapies extending beyond gastrointestinal symptom management to include systemic inflammatory conditions [28].

Methane production profoundly affects bacterial hydrogen utilization, disrupting microbial fermentation processes and leading to excessive gas accumulation [24]. In conditions like IMO, this altered fermentation further exacerbates bloating and discomfort, which significantly impacts patients’ quality of life. These effects highlight how microbial dynamics, driven by methane-producing organisms, contribute to the clinical manifestations of dysbiosis syndromes and the necessity of integrating methane detection into diagnostic protocols [24,30].

The impaired gut motility results in prolonged retention of luminal contents, creating an environment conducive to imbalances in bacterial and fungal communities. This perpetuation of dysbiosis underscores the interconnectedness of methane production, microbial imbalances, and symptoms such as constipation. Strategies to improve motility, such as prokinetics or methane-targeting therapies, may mitigate microbial overgrowth, further supporting their integration into treatment paradigms for IMO and related disorders.

Elevated methane levels detected through breath testing provide a valuable diagnostic tool for identifying methanogen-predominant conditions like IMO. However, while these tests offer insights into microbial activity, they must be complemented by other diagnostic tools to ensure accurate detection and effective treatment planning [6].

Interactions between fungi and bacteria are central to the dysbiosis syndromes of both the small and large intestines. For instance, the co-proliferation of *Candida tropicalis* and *Escherichia coli* not only exacerbates intestinal dysfunction but also highlights the complexity of microbial interactions in forming pathogenic biofilms [29,31]. These multi-species biofilms represent formidable therapeutic challenges due to their heightened resistance to antimicrobials, emphasizing the critical need for biofilm-disrupting agents in combination with conventional treatments [29,31].

Mixed infections involving fungi and bacteria often lead to overlapping symptoms, complicating the clinical presentation and delaying accurate diagnosis. Emerging molecular diagnostic techniques, such as advanced fungal and bacterial DNA sequencing, offer critical insights into these mixed infections. These tools are poised to be indispensable in guiding targeted interventions, especially for patients with complex dysbiosis syndromes [8].

Effective management of bacterial and fungal imbalances necessitates combination therapies incorporating biofilm-targeting agents alongside traditional antibiotics and antifungals. Addressing biofilms is pivotal in overcoming microbial resistance and achieving long-term remission in dysbiosis conditions. These treatment strategies signify a more holistic approach to restoring microbial equilibrium and resolving persistent symptoms [29].

Motility disorders and immune dysfunction are fundamental contributors to dysbiosis, as they facilitate the colonization of pathogenic species. Impaired intestinal transit, as observed in IBS-C, creates an environment conducive to bacterial and fungal overgrowth, while immune system dysregulation increases susceptibility to these colonizations. These physiological dysfunctions underscore the importance of addressing both gut transit and immune health to manage dysbiosis effectively [32].

Immune dysregulation, often observed in conditions like Crohn’s disease and IBS, is intricately linked to increased bacterial and fungal colonization in the gut. These findings highlight the role of immune system dysfunction in initiating and perpetuating dysbiosis, emphasizing the importance of immune-focused therapies as part of a multifaceted treatment approach [33].

The interplay between gut transit, immune pathways, and microbial composition underscores the complexity of dysbiosis syndromes and the need for multifaceted diagnostic and therapeutic strategies. For instance, variations in *Methanosphaera stadtmanae* abundance observed during IBD remission illustrate the associative relationship between microbial composition and disease state, although these findings should not be interpreted as direct treatment effects [22,25].

Molecular diagnostic advancements, such as shotgun metagenomic sequencing, facilitate the precise detection of microbial contributors to dysbiosis, including traditionally under-recognized players like methanogens and fungi. Tools such as molecular diagnostic advancements (especially shotgun metagenomic sequencing) not only improve diagnostic accuracy but also inform the development of tailored therapeutic strategies that address individual patients’ microbial profiles, promising advancements in personalized medicine [8].

Comprehensive treatment strategies combining antifungal drugs, biofilm-disrupting agents, and microbiome-based therapies, such as fecal microbiota transplantation (FMT), hold significant potential for achieving gut homeostasis and improving outcomes in dysbiosis syndromes. These interventions represent a critical step towards integrating innovative therapeutic approaches into standard care practices [28,34].

Altogether, the mechanisms driving dysbiosis syndromes are profoundly influenced by the actions of methanogens and fungi. Thorough understanding and targeted interventions can pave the way for improved clinical management and patient outcomes.

### 3.1. Microbiota-Mediated Mechanisms of the Mediterranean Diet: Implications for Intestinal and Metabolic Health

The Mediterranean diet (MD) has a significant positive influence on gut microbiota, contributing to increased microbial biodiversity and promoting the abundance of beneficial bacterial species such as *Faecalibacterium prausnitzii*, *Bifidobacterium*, and *Prevotella*, while reducing dysbiosis often associated with Western-type diets [35,36]. Its high content of dietary fibers, particularly microbiota-accessible carbohydrates (MACs), enhances the production of short-chain fatty acids like butyrate, which support gut barrier integrity, improve metabolic health, and may reduce the risk of colorectal cancer [35,36]. Moreover, polyphenols from sources such as olive oil and red wine exhibit antioxidant, anti-inflammatory, and antimicrobial properties, modulate microbial composition, and may contribute to neuroprotection via the gut–brain axis [36].

The MD’s content of polyunsaturated fatty acids (especially omega-3) supports cardiovascular and immune health by modulating gut microbiota and improving intestinal barrier function. Mediterranean diet adherence is consistently associated with increased α-diversity (greater bacterial species richness), a key indicator of a healthy gut microbiome. While strong associations exist between MD, microbiota composition, and occurrence of diseases, establishing direct cause-effect relationships often remains challenging due to the complexity and variability of microbiota responses [36].

What is certain is that minerals and micronutrients (e.g., magnesium, calcium, zinc, selenium, and vitamin B6) found in plant-based components of the MD influence microbial diversity and intestinal health through both direct and synergistic dietary effects. In contrast, the low intake of refined carbohydrates, saturated fats, and trans fats in the MD helps prevent gut dysbiosis, gut wall permeability (“leaky gut”), and associated metabolic disturbances commonly linked to the Western diet. Thus, the MD exerts its health-promoting effects, in part, through favorable modulation of the gut microbiota [35].

Additionally, the use of probiotics in MAFLD (Metabolic Associated Fatty Liver Disease) patients, by modulating the gut microbiota, led to a significant decrease in intrahepatic fat fraction, greater reductions in triglyceride levels, and improvements in liver enzymes including ALT, AST, and GGT, indicating enhanced liver function and metabolic health [37].

*Pistacia lentiscus*, or the mastic tree, is an evergreen shrub native to the Mediterranean region, especially Crete, where its resin (mastiha) has long been used in traditional medicine and as part of the Mediterranean diet. A randomized, placebo-controlled trial by Papada et al. demonstrated that supplementation with Chios mastic gum (CMG) (2.8 g/day for 3 months) in patients with endoscopy-confirmed ulcerative colitis (UC) or Crohn’s disease (CD) improved quality of life and reduced disease activity (Harvey-Bradshaw Index). Notably, in UC patients, mastiha also led to favorable changes in inflammatory (fecal lysozyme, fibrinogen) and biochemical markers (serum iron, glucose), effects not seen in the placebo group [38]. Amerikanou et al. investigated the effects of CMG on IL-17A serum levels and fecal metabolomic profiles in patients with IBD. In a randomized, placebo-controlled trial involving 129 patients with UC or CD, either in remission or relapse, participants received 2.8 g/day of CMG or placebo for 3 to 6 months. CMG supplementation significantly increased IL-17A levels and modulated fecal metabolites, notably increasing glycine and tryptophan—compounds associated with anti-inflammatory and immunoregulatory functions [38]. These findings support CMG’s role in regulating Th17 cell function in quiescent IBD. Given the involvement of IL-17A and Th17 cells in maintaining mucosal barrier integrity and controlling bacterial populations, CMG may also support host defenses against bacterial overgrowth and warrants further investigation in the context of SIBO.

### 3.2. Mediterranean Diet in Irritable Bowel Syndrome and Dysbiosis

The MD possesses several characteristics that may support gut health. Its rich content of phenolic compounds has demonstrated anti-inflammatory effects, such as the reduction in inflammatory molecule expression. Additionally, adherence to the MD is linked to a higher presence of microbiota that produce short-chain fatty acids, which are important for maintaining the integrity and function of the intestinal lining [39]. Moreover, it is observed that the MD can dynamically modulate the intestinal microbiome composition. MD adherence rates are proportional to the microbiome variations [39].

Although the generally favorable characteristics of the MD with its anti-inflammatory features and increased consumption of fiber may suggest that this diet would be a suitable match for IBS patients, it is not certain. Chen et al.’s study found no overall difference in MD adherence between IBS patients (106 patients) and healthy controls (108 patients), nor a correlation between MD adherence and IBS symptoms. However, specific food items revealed unexpected associations—some typically healthy (pro-MD) foods like cantaloupe were linked to worse symptoms, while some unhealthy (anti-MD) foods like soda were linked to milder symptoms [40]. In contrast, Zito et al. conducted a multivariate analysis that indicates that patients with IBS are significantly more likely to have low or intermediate adherence to the MD compared to those with high adherence. Specifically, IBS and functional dyspepsia were both associated with low MD adherence, while intermediate adherence was also linked to IBS. These findings suggest that greater adherence to the Mediterranean diet may have a protective effect against IBS [41]. The effect of the MD on IBS and general dysbiosis treatment remains unclear and requires further research.

### 3.3. The Role of Laboratory Analysis in the Assessment of Gut Microbiota Composition

Laboratory analysis plays a crucial role in evaluating gut microbiota composition by offering precise and quantifiable insights into the diversity and abundance of microbial species. Techniques such as 16S rRNA gene sequencing, 18S rRNA gene sequencing, ITS, and metagenomic analysis enable the identification of even low-abundance microorganisms that may have significant clinical relevance [42,43].These advanced methods allow for comprehensive microbiome characterization and facilitate the exploration of associations between microbial patterns and health outcomes. Without laboratory-based approaches, such assessments would remain speculative and lack the scientific rigor required for diagnostic or therapeutic decision-making.

In the context of SIBO, small bowel aspirate and culture—detecting bacterial growth between 10^3^ and 10^5^ CFU/mL—is considered the gold standard for diagnosis, despite its invasive nature limiting routine use [44]. A study using duodenal aspirate and culture demonstrated that individuals with small intestinal dysmotility were at significantly greater risk of SIBO. Specifically, when using a > 10^3^ CFU/mL threshold, the odds ratio (OR) was 3.6 (*p* = 0.0003), while for a >10^5^ CFU/mL threshold, the OR was 2.7 (*p* = 0.005) [45,46]. Moreover, a markedly higher proportion of patients with both IBS and SIBO exhibited signs of dysmotility compared to those with IBS alone (86% vs. 39%, respectively; *p* = 0.02) [46]. These findings underscore the indispensable role of laboratory diagnostics in accurately identifying microbial imbalances such as SIBO.

### 3.4. Clinical Assessment

A thorough understanding of clinical assessment is critical for diagnosing gastrointestinal dysbiosis syndromes, including SIBO, IMO, and SIFO. By examining various diagnostic methods and treatment approaches, this section will elucidate the complexities of identifying microbial imbalances and the strategies employed to manage them effectively. Readers will gain insight into how combining innovative technologies with traditional practices can enhance diagnostic accuracy and patient outcomes, reinforcing the necessity of a comprehensive approach to addressing these multifaceted conditions.

Hydrogen and methane breath tests are among the most frequently employed non-invasive diagnostic tools for identifying SIBO and IMO. These tests measure exhaled gas concentrations after ingesting carbohydrate substrates such as glucose or lactulose. An increase in hydrogen levels of 20 ppm or more above baseline within 90 min of administration confirms SIBO, while methane levels of 10 ppm or higher at any time are considered diagnostic for IMO [6]. The widespread utilization of breath tests highlights their accessibility and ease of use, lending them considerable clinical utility. However, concerns regarding their variability in sensitivity and specificity have been extensively documented. Lactulose breath tests, for instance, demonstrate a sensitivity range of 31% to 68% and specificity from 44% to 100%, indicating an elevated risk of both false positives and negatives, particularly in scenarios involving distal small intestine overgrowth or rapid intestinal transit [4,7]. Despite these limitations, their non-invasive nature makes them especially valuable for patients unable or reluctant to undergo invasive procedures, such as small bowel aspiration.

The primary advantage of hydrogen and methane breath tests lies in their widespread availability and overall safety profile. They represent a practical diagnostic option for diverse patient populations, particularly those with contraindications for invasive diagnostic methods. However, their diagnostic accuracy is compromised by several factors, including patient preparation, substrate selection, and the overlapping fermentation processes in the colon. For example, using glucose as a substrate, while advantageous for its rapid absorption in the proximal small intestine, often fails to detect overgrowth located in the distal sections [7]. Furthermore, lactulose, though capable of reaching the distal small intestine, is prone to producing false positives due to its fermentation in the colon. These technical challenges underscore the need for refined protocols or adjunctive diagnostic methods to enhance the reliability of breath tests.

Small-bowel aspiration and culture, particularly using the Rao technique during upper esophagogastroduodenoscopy, is often regarded as the gold-standard diagnostic tool for SIBO. This method involves obtaining direct samples from the duodenum or jejunum for microbial analysis, with bacterial counts exceeding 10^3^ CFU per milliliter confirming overgrowth. Alternative thresholds, such as 10^5^ CFU/mL, have been proposed to improve diagnostic precision, particularly in addressing variations in microbial loads among individuals [7,21]. While significantly more accurate than breath tests, small-bowel aspiration is hindered by its invasiveness, high costs, and the need for specialized microbiological facilities, limiting its routine use. Practical barriers, such as the risk of sample contamination and difficulty obtaining representative samples from the distal small intestine, pose further challenges to its broader clinical application. Consequently, this method is typically reserved for complex or inconclusive cases where non-invasive tests fail to provide definitive results.

Molecular diagnostic techniques, such as next-generation sequencing and shotgun metagenomic sequencing, represent notable advancements in detecting microbial populations implicated in dysbiosis conditions such as SIBO, IMO, and SIFO. These methods enable the detailed identification of bacterial and fungal species, including *Methanobrevibacter smithii* and *Candida albicans*, thereby offering significant precision compared to conventional diagnostic approaches [22]. Molecular tools have effectively guided targeted therapeutic strategies, particularly in complex or multifactorial dysbiosis syndromes where mixed bacterial and fungal overgrowth coexist. However, these techniques are often cost-prohibitive and face technical limitations that currently restrict their widespread adoption. They also require standardization across laboratories to ensure reliability and consistency in clinical practice [47]. Despite these challenges, their integration into comprehensive diagnostic protocols holds promise for addressing the limitations of traditional diagnostic methods like breath testing.

The diagnostic challenges associated with fungal overgrowth, particularly SIFO, further underscore the need for advanced methodologies. Traditional fungal culture techniques often fail to adequately identify pathogenic fungal species due to their limited sensitivity and specificity. Emerging methods such as fungal DNA sequencing and duodenal aspirate culture have improved diagnostic accuracy by directly identifying fungal species and assessing their contribution to mucosal inflammation [28,47]. Despite these advancements, the underdiagnosis of fungal dysbiosis remains a significant clinical problem, driven by a lack of routine testing in conventional practice. Incorporating fungal diagnostics into standard evaluations of dysbiosis is essential for optimizing treatment strategies, particularly given the symptomatic overlap between fungal and bacterial overgrowth.

Long-term PPI use is a well-documented risk factor for SIBO, as PPIs reduce gastric acidity, thereby impairing natural defense mechanisms against bacterial colonization in the small intestine. Studies indicate an increased prevalence of SIBO among PPI users, with rates reaching up to 72% [45]. This association necessitates careful consideration of PPI use when interpreting diagnostic findings, as altered gut physiology due to suppressed gastric acid may complicate breath test results or other diagnostic methods [48]. To address this, tailored diagnostic protocols that account for the effects of PPI therapy are essential. Combining molecular diagnostics with breath testing or small-bowel aspiration could enhance diagnostic accuracy in this subset of patients, ensuring more precise identification of PPI-associated dysbiosis [8].

A multimodal approach to diagnosis is increasingly recognized as essential for improving the accuracy of dysbiosis detection. By integrating breath tests with molecular diagnostics and invasive techniques such as aspiration and culture, clinicians can address the respective limitations of each method [49,50]. This combined methodology is particularly valuable in cases involving overlapping symptoms of SIBO, IMO, and SIFO, where reliance on a single diagnostic modality may yield inconclusive or misleading results. For instance, molecular diagnostics can complement breath tests by identifying specific microbial contributors, such as methanogens or pathogenic fungi, that are challenging to detect through conventional means [51]. This approach reduces the risk of misdiagnosis and facilitates more targeted treatment strategies.

Future research should prioritize the development of comprehensive diagnostic protocols that integrate these diverse methodologies while also addressing practical considerations such as cost, accessibility, and procedural feasibility [50]. Such advancements can significantly improve clinical outcomes by enabling more precise identification of dysbiosis-related conditions. Beyond gastrointestinal disorders, these integrated diagnostic frameworks may have broader applications, as demonstrated in cancer research. For instance, multi-omics approaches combining genomics, proteomics, and metabolomics have been instrumental in understanding microbiome-host interactions and personalizing cancer therapies [52]. Incorporating similar techniques into dysbiosis diagnostics could further enrich the field and provide valuable insights into microbial contributions to systemic health.

In conclusion, diagnostic methods for conditions like SIBO, IMO, and SIFO must evolve to address the inherent limitations of existing approaches. Integrating non-invasive, invasive, and molecular techniques offers the most promise for achieving diagnostic accuracy and informing individualized treatment strategies. Continued advancements in this area are critical for improving patient outcomes and expanding the understanding of gastrointestinal dysbiosis.

### 3.5. Treatment Approaches

Treatment approaches for gastrointestinal dysbiosis syndromes are diverse, encompassing a range of pharmacological, dietary, and microbiome-modulating strategies tailored to address the underlying microbial imbalances. Antibiotic therapies form the cornerstone of management for SIBO and IMO. Rifaximin, a minimally absorbed antibiotic, stands out with documented efficacy rates ranging between 61% and 78% for SIBO treatment [6]. However, recurrence remains a significant challenge, with nearly 43.7% of SIBO patients experiencing symptom relapse within nine months of treatment. This high recurrence rate underscores the need for alternative or adjunctive strategies for long-term symptom control and stability. Comparative studies reveal that antibiotic treatment yields symptomatic improvement in 49.5% of SIBO patients, a stark contrast to the 13.7% improvement observed in untreated individuals, with a calculated number needed to treat (NNT) of 2.8 [53]. While rifaximin demonstrates high efficacy, its benefits often diminish over time due to the persistence of predisposing factors for dysbiosis, highlighting the necessity for an integrated treatment framework beyond antibiotics alone.

Combination antibiotic therapy offers an enhanced approach for specific dysbiosis profiles, particularly for methane-dominant IMO. Rifaximin combined with neomycin has demonstrated clinical superiority in eradicating methane-producing archaea compared to monotherapy [6]. This synergistic approach addresses the metabolic resilience of methanogens, which pose challenges to single-agent antibiotic regimens. Methanogens such as *Methanobrevibacter smithii* exhibit unique metabolic pathways that necessitate targeted therapeutic interventions, reinforcing the rationale for combination therapies. However, the emergence of antibiotic resistance, coupled with the negative impact of broad-spectrum treatments on commensal microbial populations, raises critical questions about the long-term sustainability of such approaches. Strategies to mitigate these complications, such as the addition of probiotics or alternative therapies, require further exploration to reduce reliance on antibiotics while preserving their efficacy. Rifaximin’s safety profile and demonstrated success in other gastrointestinal conditions, like hepatic encephalopathy, underscore its versatility as a treatment option, but further research is needed to bridge the gap in its sustained efficacy for dysbiosis-related syndromes [54].

In summary, the combination of rifaximin and neomycin is one of the most effective treatment regimens for small intestinal bacterial overgrowth with dominant methane production.

Adjunctive therapies, including probiotics, offer promising avenues to reduce recurrence and enhance microbial equilibrium following antibiotic treatments. Probiotic administration aims to restore dysbiosis-induced imbalances by replenishing beneficial bacterial populations while countering the disruptive effects of antibiotic overuse on gut microbiota. While some studies have demonstrated that probiotics reduce recurrence rates of SIBO and improve symptom management, inconsistencies in strain selection, dosage, and duration pose challenges to their widespread clinical adoption [8,55]. Targeted research is essential to identify optimal microbial strains with specific therapeutic benefits, ensuring that probiotics achieve tangible outcomes without exacerbating fermentation and other dysbiosis-related symptoms. Studies also suggest that restoring microbial diversity and addressing particular bacterial populations, such as those identified through bacterial culture techniques, may enhance probiotic efficacy. For instance, it has been observed that SIBO patients often show an increased prevalence of *Streptococcus* species and a lower abundance of *Bacteroides*, signaling the need to target these patterns of microbial imbalance during treatment [56]. An optimal therapeutic strategy should focus on the administration of rifaximin at a dosage of 1600 mg daily for two weeks, combined with appropriately selected probiotic therapy containing *Saccharomyces boulardii* strains. Combining probiotics with rifaximin or other antibiotics represents a pragmatic approach to counteract dysbiosis-related complications [56,57]. Although probiotics (for example, such as the mentioned *S. boulardii)* have demonstrated potential benefits in the management of dysbiosis, the precise strains, optimal dosages, and commercial formulations have not yet been sufficiently evaluated to establish evidence-based therapeutic guidelines [57]. Additionally, further clarification is necessary regarding the potential for antagonistic interactions between probiotics and conventional therapies.

Exclusive elemental diets (EDs) have shown significant efficacy as a non-pharmacological intervention for dysbiosis syndromes, particularly hydrogen-dominant SIBO. These diets, consisting of pre-digested nutrients that minimize microbial fermentative activity, have demonstrated normalization of breath test results in 80% of patients within 14 days, with an additional 5% achieving normalization after a 21-day extension [58]. Symptomatic relief results from the reduction in fermentable substrates, which limits the resources available for pathogenic bacteria and decreases gas production. Elemental diets also exhibit utility in addressing methane levels in IMO, as evidenced by clinical trials reporting marked reductions in exhaled methane and hydrogen levels following dietary intervention [58]. While their efficacy is well-documented, the restrictive nature of elemental diets and their potential impact on patients’ nutritional status raise concerns about long-term adherence and sustainability. The utility of elemental diets lies in their ability to achieve rapid microbial shifts, but their integration with other therapeutic strategies is crucial for maintaining microbial balance post-intervention.

Building on this concept of dietary modulation, probiotics and prebiotics represent a complementary approach that directly targets the composition and function of the gut microbiota, focusing on rebalancing the gut ecosystem to mitigate symptoms and restore equilibrium. Probiotics, because of their ability to promote beneficial changes in microbiota composition, show potential for symptom alleviation in dysbiosis, but their standalone effectiveness for conditions like SIBO and IMO remains inconsistent [8,55]. Prebiotics, in contrast, serve as substrates for beneficial microorganisms, selectively enhancing their growth while suppressing pathogenic populations. However, excessive prebiotic use risks aggravating symptoms such as bloating due to over-fermentation, illustrating the need for precision in therapeutic application. The simultaneous use of probiotics and prebiotics, commonly referred to as synbiotics, represents an integrated strategy to optimize microbial rebalancing, particularly in cases of treatment-resistant dysbiosis. Nevertheless, comprehensive studies are needed to establish standardized protocols for strain selection and dosing to ensure consistent efficacy.

While probiotics and prebiotics primarily aim to restore bacterial balance, antifungal therapies remain indispensable when fungal overgrowth, such as SIFO, is the dominant driver of dysbiosis. Fluconazole and nystatin are commonly employed agents targeting fungal pathogens like *Candida albicans*. The effectiveness of these treatments depends heavily on accurate fungal identification through advanced diagnostics, such as fungal DNA sequencing, which has facilitated more precise fungal pathogen detection and corresponding therapeutic interventions [8,51]. However, fungal biofilms present a substantial barrier to successful treatment, as these structures enhance microbial resistance and complicate pathogen clearance. Biofilm-forming fungi, such as *Candida albicans*, are particularly adept at damaging the gastrointestinal mucosa and exacerbating inflammatory responses, further underscoring the need for targeted interventions [56]. Adjunctive therapies incorporating biofilm-disrupting agents into antifungal protocols have demonstrated improved outcomes by enhancing drug penetration and reducing resistance [59]. These strategies underscore the necessity of integrating biofilm-focused agents into managing fungal dysbiosis to achieve sustained microbial clearance and symptomatic resolution.

Fecal microbiota transplantation (FMT) has emerged as an innovative therapeutic option with significant potential for restoring microbial balance in dysbiosis syndromes. As widely used for *Clostridioides difficile* infections, FMT boasts efficacy rates of 85% of recurrent *Clostridium difficile* infections and 55% of new *Clostridioides difficile* infections and is a promising intervention for SIBO, IMO, and SIFO [34,60]. Beyond resolving bacterial dysbiosis, FMT offers additional benefits, such as reducing colonic inflammation and enhancing mucosal immunity, which broadens its therapeutic scope. FMT’s potential to address systemic microbial imbalances and modulate immune responses highlights its utility as a customizable treatment for complex dysbiosis syndromes. However, tailoring FMT for conditions involving methanogens or fungi remains underexplored with substantial research opportunities. Integrating FMT with other microbiome-based therapies may further enhance its efficacy, but safety and donor standardization remain critical concerns that require ongoing investigation.

Biofilm formation represents a critical therapeutic challenge in treating dysbiosis-related syndromes complicated by bacterial and fungal overgrowth. Pathogenic biofilms, such as those formed by *Candida tropicalis* and *Escherichia coli*, exacerbate microbial persistence and resistance to conventional antimicrobials, necessitating specialized interventions [59,61]. Agents specifically targeting biofilm structures, including enzymes and antimicrobial compounds, have demonstrated utility in disrupting these resilient microbial communities and enhancing treatment response.

Therapeutic strategies targeting immune pathways are an emerging area of interest. These include the use of biologics and small molecules that modulate inflammatory cytokines (e.g., TNF-α, IL-6, IL-17), many of which are already used in conditions such as inflammatory bowel disease. While such treatments are not yet standard for SIBO or related dysbiosis syndromes, early evidence suggests that regulating immune responses could help break the cycle of inflammation and dysbiosis, particularly in treatment-resistant or recurrent cases.

Additionally, some novel approaches aim to modulate the immune system indirectly by targeting the gut microbiota itself through postbiotics, engineered probiotics, or immunobiotics that can enhance regulatory T-cell activity and restore mucosal tolerance. However, these therapies are still largely experimental, and more clinical studies are needed to define their role in dysbiosis treatment.

In conclusion, by addressing the specific microbial imbalances underlying each condition, these targeted interventions hold promise for improving clinical outcomes and reducing recurrence. Table 1 below differentiates the individual therapies mentioned in this article.

## 4. Conclusions

By summarizing and contextualizing these findings, the work underscores its contributions to advancing the understanding of dysbiosis syndromes while recognizing the need for continued innovation to overcome current limitations.

Across the literature, several critical gaps persist: the lack of standardized diagnostic protocols that integrate non-invasive and molecular tools; limited understanding of the interplay between bacteria, archaea, and fungi in shaping dysbiosis trajectories; insufficient data on long-term outcomes of microbiome-modulating therapies; and insufficient personalization of interventions based on individual microbial profiles.

**Figure 1:**
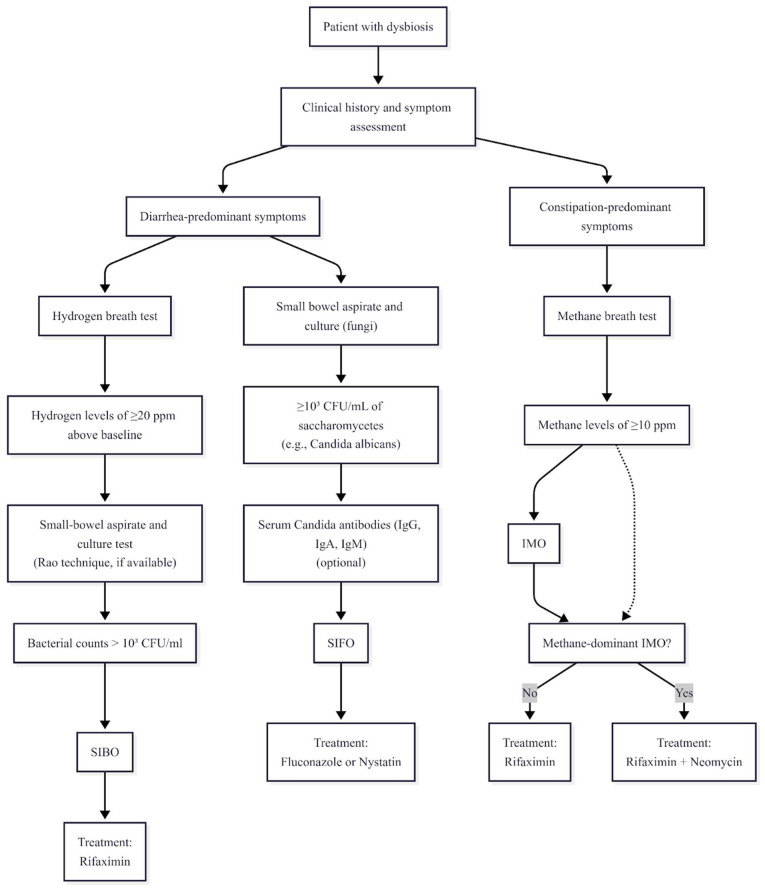
Diagnostic and therapeutic algorithm.

Future research must therefore prioritize harmonizing diagnostic criteria, advancing cost-effective sequencing-based tools, and developing standardized biofilm-targeting and microbiome-restoring interventions. A proposed diagnostic and therapeutic algorithm is illustrated in Figure 1. Importantly, integrating personalized medicine approaches—tailoring therapy according to microbial composition, host genetics, and clinical phenotype—represents the most promising pathway toward durable disease control. Such a shift will not only improve gastrointestinal outcomes but may also reshape systemic health management, given the growing recognition of gut dysbiosis as a driver of extra-intestinal disease.

## Figures and Tables

**Table 1:** Treatment approaches for gastrointestinal dysbiosis syndromes.

Therapy	Target Condition	Mechanism of Action	Efficacy	Limitations/Considerations
**Rifaximin**	SIBO, IMO	Minimally absorbed antibiotic; reduces bacterial overgrowth [6,53]	61–78% efficacy in SIBO; NNT = 2.8 [53]	High recurrence (43–70%); ↑ resistance; reduced long-term efficacy
**Rifaximin + Neomycin**	Methane-dominant IMO	Synergistic antibiotic combination; targets methane-producing archaea [6,62]	More effective than monotherapy for methane eradication	Risk of resistance; impacts on commensal flora
**Fluconazole/Nystatin**	SIFO	Antifungal agents; inhibit fungal cell membrane synthesis	Effective against *Candida albicans* and other fungi [17,53]	Requires accurate diagnosis; antifungal resistance is a concern [8,52,63,64]
**Biofilm-Disruptive Agents**	Biofilm-associated SIBO/SIFO	Enzymes or compounds targeting biofilm matrices	Enhance mucolysis, penetration, and efficacy [59,61]	Still under investigation; requires integration with main therapy [59,61]
**Elemental Diet**	Hydrogen-dominant SIBO, IMO	Starves fermentative bacteria; reduces complex substrates	Normalization of breath tests in ~80% after 14 days [58]	Poor palatability; long-term sustainability concerns [6,58]
**Probiotics**	Post-antibiotic dysbiosis, SIBO, IMO	Replenish beneficial bacteria; reduce pathogen load	May reduce recurrence and improve outcomes [8,55]	Variable outcomes; strain- and dose-dependent inconsistencies [8,14,38,63,64]
**Prebiotics**	General dysbiosis	Encourage growth of beneficial microbes	Modulate microbiota composition	May worsen symptoms due to fermentation [55]
**Synbiotics (Probiotics + Prebiotics)**	Resistant dysbiosis	Combined substrate + strain	Promising for microbial restoration	Lacks standardized protocols [55]
**Fecal Microbiota Transplantation (FMT)**	SIBO, IMO, SIFO (recurrent)	Restores diverse and balanced microbiota	Efficacy of 85% for recurrent *C. difficile* infections and 55% for new *C. difficile* infections [60]; potential in SIBO/dysbiosis	Donor variability, safety concerns, limited data for SIBO/IMO [8,34,65]
**Vancomycin/Fidaxomicin/Metronidazole**	*Clostridioides difficile* infection (CDI)	Inhibit bacterial cell wall synthesis or DNA function	Fidaxomicin: similar initial cure rates to vancomycin but lower recurrence (< 50%) [63,64]; vancomycin: superior to metronidazole, especially in severe CDI [63,64]	CDI may recur; secondary dysbiosis; use if recurrence is common [63,64]

## Data Availability

No new data were created or analyzed in this study. Data sharing is not applicable to this article.
